# Joint Involvement in Primary Sjögren's Syndrome: An Ultrasound “Target Area Approach to Arthritis”

**DOI:** 10.1155/2013/640265

**Published:** 2013-07-08

**Authors:** Luis M. Amezcua-Guerra, Fritz Hofmann, Angelica Vargas, Pedro Rodriguez-Henriquez, Carla Solano, Cristina Hernández-Díaz, Diana Castillo-Martinez, Lucio Ventura-Ríos, Marwin Gutiérrez, Carlos Pineda

**Affiliations:** ^1^Department of Immunology, Instituto Nacional de Cardiología Ignacio Chávez, Juan Badiano 1, Sección XVI, Tlalpan, 14080 Mexico City, Mexico; ^2^Department of Musculoskeletal Ultrasonography, Instituto Nacional de Rehabilitación, Avenue México-Xochimilco 289, Arenal de Guadalupe, Tlalpan, 14389 Mexico City, Mexico; ^3^Department of Rheumatology, Instituto Nacional de Cardiología Ignacio Chávez, Juan Badiano 1, Sección XVI, Tlalpan, 14080 Mexico City, Mexico; ^4^Department of Rheumatology, Hospital General Dr. Manuel Gea González, Calzada de Tlalpan, 4800, Sección XVI, Tlalpan, 14080 Mexico City, Mexico; ^5^Department of Rheumatology, Hospital Nacional Rosales and Instituto Salvadoreño del Seguro Social, Final calle Arce 25 Avenue Norte, San Salvador, El Salvador; ^6^Department of Dermatology, Hospital General de Zona 1-A Dr. Rodolfo Antonio de Mucha Macías, Instituto Mexicano del Seguro Social, Municipio Libre 270, Portales Sur, Benito Juarez, 03300 Mexico City, Mexico; ^7^Clinica Reumatologica, Università Politecnica delle Marche, Via dei Colli 52, Jesi, 60035 Ancona, Italy

## Abstract

*Objective*. To characterize the ultrasound (US) pattern of joint involvement in primary Sjögren's syndrome (pSS). *Methods*. Seventeen patients with pSS, 18 with secondary Sjögren's syndrome (sSS), and 17 healthy controls underwent US examinations of various articular regions. Synovitis (synovial hypertrophy/joint effusion), power Doppler (PD) signals, and erosions were assessed. *Results*. In patients with pSS, synovitis was found in the metacarpophalangeal joints (MCP, 76%), wrists (76%), and knees (76%), while the proximal interphalangeal joints, elbows, and ankles were mostly unscathed. Intra-articular PD signals were occasionally detected in wrists (12%), elbows (6%), and knees (6%). Erosions were evident in the wrists of three (18%) patients with pSS, one of these also having anti-cyclic citrullinated peptide (anti-CCP) antibodies. While US synovitis does not discriminate between sSS and pSS, demonstration of bone erosions in the 2nd MCP joints showed 28.8% sensitivity and 100% specificity for diagnosing sSS; in comparison, these figures were 72.2 and 94.1% for circulating anti-CCP antibodies. *Conclusions*. In pSS, the pattern of joint involvement by US is polyarticular, bilateral, and symmetrical. Synovitis is the US sign most commonly found in patients with pSS, especially in MCP joints, wrists, and knees, and bone erosions also may occur.

## 1. Introduction

Primary Sjögren's syndrome (pSS) is a systemic autoimmune disorder characterized by diffuse lymphocytic infiltration of epithelia. Involvement of salivary and lachrymal glands and their clinical counterparts, xerostomia and xerophthalmia, are the key clinical hallmarks [1]. Musculoskeletal manifestations such as myalgias, morning stiffness, and arthralgias are present in as many as 90% of patients and arthritis in up to 17% [1, 2]. When followed for >50 months, at least 18% of patients with pSS will develop polyarticular arthritis [3]. Thus, patients with pSS may present with chronic arthritis that clinically resembles rheumatoid arthritis (RA). However, the erosive nature and distribution of joint involvement in pSS as distinguishing features remain controversial. Some studies based on conventional X-rays of the hands have shown nonerosive arthritis, leading them to consider it as a mild and nonaggressive oligoarthritis [4, 5]. In contrast, others demonstrated the presence of severe polyarthritis with features resembling RA, including erosions, particularly in patients with antibodies against cyclic citrullinated peptides (anti-CCP) and selected alleles of major histocompatibility complex (MHC) class II molecules [6, 7].

Over the past decade, there has been a growing interest in rheumatology to implement ultrasound (US) as an instrument for the routine assessment of different rheumatic conditions [8, 9]. When compared with X-rays, US is more sensitive to identify bone erosions in patients with various forms of arthritis including spondyloarthritis, RA, and systemic lupus erythematosus (SLE) [10–15]. In this context, US may represent a useful imaging technique to elucidate the paradigm of the nonerosive nature of joint involvement in inflammatory arthritides, including pSS.

To date, few studies aimed at characterizing the US pattern of joint involvement in patients with pSS have been conducted [16–18]. These are consistent in demonstrating significant involvement targeting the synovial membrane as featured by synovial hypertrophy, joint effusion, and intra-articular power Doppler (PD); however, they provide no strong data on the existence of bone erosions.

The aim of the present study was to characterize the US pattern of joint involvement in patients with pSS, with an emphasis on synovitis and bone erosions. The ability of US to discriminate between pSS and RA-associated secondary Sjögren's syndrome (sSS) also was assessed.

## 2. Materials and Methods

### 2.1. Patients

The study was conducted in consecutive patients with an established diagnosis of pSS according to the American-European Consensus Group (AECG) criteria [19]. Age- and gender-matched patients with an established diagnosis of RA according to the 2010 American College of Rheumatism/European League Against Rheumatism (ACR/EULAR) classification criteria for RA, and also fulfilling the AECG criteria for SS, were included as a disease control group [20]. Additionally, age- and gender-matched individuals with neither *sicca* syndrome nor clinical data suggestive of RA or pSS were included as a “healthy” control group.

All patients were attending the Rheumatology Outpatient Clinic at the Instituto Nacional de Cardiología, Mexico City, Mexico. Patients with inflammatory arthropathies other than RA, a history of malignancy, clinical suspicion of infection, hand surgery, or age <18 years were not included. Prior to the US assessment, both patients with pSS and those with sSS underwent a detailed clinical history by two experienced rheumatologists (AV, LMA-G) who did not participate in the US examinations. All patients had standard X-rays of hands performed on the same day that the US images were acquired. Medical, laboratory, and imaging data were obtained according to a standardized *pro forma* file.

The study was conducted in accordance with the Declaration of Helsinki and local regulations. The Ethics Committees of the Instituto Nacional de Cardiología and the Instituto Nacional de Rehabilitación approved the study protocol. All participants signed an informed consent format.

### 2.2. Laboratory Assessment

Five mL of peripheral blood was obtained from all participants on the same day that the US study was performed. Samples were centrifuged (3,000 rpm, for 15 min, at 3°C), and sera were stored in aliquots at –70°C until used. Serum C-reactive protein (reference value, <9.2 mg/L) and IgM rheumatoid factor (RF, <20 IU/mL) were analyzed by nephelometry (Beckman Coulter IMMAGE, Brea, CA, USA). Antinuclear antibodies (ANA) were detected by indirect immunofluorescence on HEp-2 cell slides (Inova Diagnostics, San Diego, CA, USA), and any serum sample showing fluorescence at a dilution >1 : 80 was considered positive. Anti-Ro/SSA, anti-La/SSB (Inova), and anti-CCP antibodies (2nd generation assay; Euroimmun, Lübeck, Germany) were detected by semi-quantitative enzyme-linked immunosorbent assays (ELISA). Results were analyzed according to the cutoff values provided by each manufacturer and reported as positive or negative.

### 2.3. Ultrasound Assessment

US examinations were performed with a Sonoline Antares (Siemens, Erlanger, Germany), equipped with a 7–13 MHz broadband, a multifrequency linear array transducer (axial resolution = 0.19 [–12 dB (mm)], and lateral resolution = 0.43 [–12 dB (mm)]). All scans were performed by a rheumatologist (PR-H) trained in musculoskeletal US who was blinded to clinical, radiographic, and laboratory data. US examinations were performed at random, and patients were asked not to discuss their medical condition with the sonographer. Representative images were acquired and digitally recorded, and an electronic file was created for each patient. All recorded images were interpreted together by a second experienced sonographer (CP), who was also blinded to clinical and laboratory data. Agreement between sonographers was assessed, and discrepancies were solved by consensus.

All US examinations were performed using a multiplanar scanning technique according to EULAR guidelines for musculoskeletal US in rheumatology [21]. The following anatomical areas were scanned bilaterally: 1st–5th metacarpophalangeal joints (MCP); 1st interphalangeal (IP) joint; 2nd–5th proximal interphalangeal joints (PIP); radiocarpal and midcarpal joints (wrist); and elbow, knee, and ankle joints.

Initially, each joint was scanned in grey scale to detect morphostructural changes and subsequently with the PD technique to detect intra-articular blood perfusion. Blood flow assessment was performed with settings standardized as follows: pulse repetition frequency = 610 Hz; Doppler frequency = 6–8.9 MHz; and wall filter = 4. PD gain was adjusted to avoid the display of random noise. For assessment of small joints, a plentiful quantity of gel was used and care was taken not to compress tissues under examination to avoid “blanching” of the PD signal due to transducer pressure.

### 2.4. Ultrasound Interpretation

For US elementary lesions, Outcome Measures in Rheumatology (OMERACT) preliminary definitions were adopted [22]. The following abnormalities were recorded: synovitis; bone erosions and presence of intra-articular PD signal. Synovitis was defined as the presence of either synovial hypertrophy or joint effusion, or both. Synovial effusion was defined as abnormal hypo- or anechoic (relative to subdermal fat) intra-articular material that is displaceable and compressible, but that does not exhibit a PD signal. Synovial hypertrophy was defined as abnormal hypoechoic (relative to subdermal fat) intra-articular tissue that is nondisplaceable and poorly compressible and that may exhibit a PD signal. Bone erosion was defined as definite intra-articular cortical interruption with a step-down contour defect visible in both longitudinal and transverse views. Any intra-articular PD signal was considered pathological. A binary score (0 = absent; 1 = present) was used to assess the presence of synovitis, bone erosions, and PD signals. In wrists, the involvement was considered present when at least one of the radiocarpal or midcarpal joints displayed one elementary lesion. 

### 2.5. Statistical Analysis

Frequencies and proportions were utilized to describe categorical data, and differences were analyzed using the Fisher exact test. Sensitivity and specificity were calculated in 2 × 2 tables. Continuous variables were expressed as median (minimum-to-maximum range) and compared using the Mann-Whitney test. All analyses were two tailed, and significance was set at *P* < 0.05. Graph Pad Prism ver.4.02 (Graph Pad Software, San Diego CA, USA) statistical software was employed for the calculations.

Interobserver agreement was assessed by the Cohen unweighted *κ* test, while symmetry of joint involvement was calculated according to the Wilson efficient-score method corrected for continuity (observed/expected by chance) on the VassarStats Website for Statistical Computation [23].

## 3. Results

The study was conducted on 17 female patients with pSS, 18 patients with sSS, and 17 control subjects. Median age of patients with pSS was 58 years (range, 32–83 years) with median disease duration of 3 years (range, 1–18 years) whereas, for patients with sSS, median age was 61 years (range, 40–77 years), and median disease duration was 9 years (range, 1–30 years). Median age of controls was 58 years (range, 32–58 years). Clinical and demographic data are described in Table 1. As noted, patients with sSS had longer disease duration and higher frequency of positive anti-CCP antibodies, whereas anti-Ro/SSA and anti-La/SSB antibodies were more frequent in pSS patients. 

**Figure 1:**
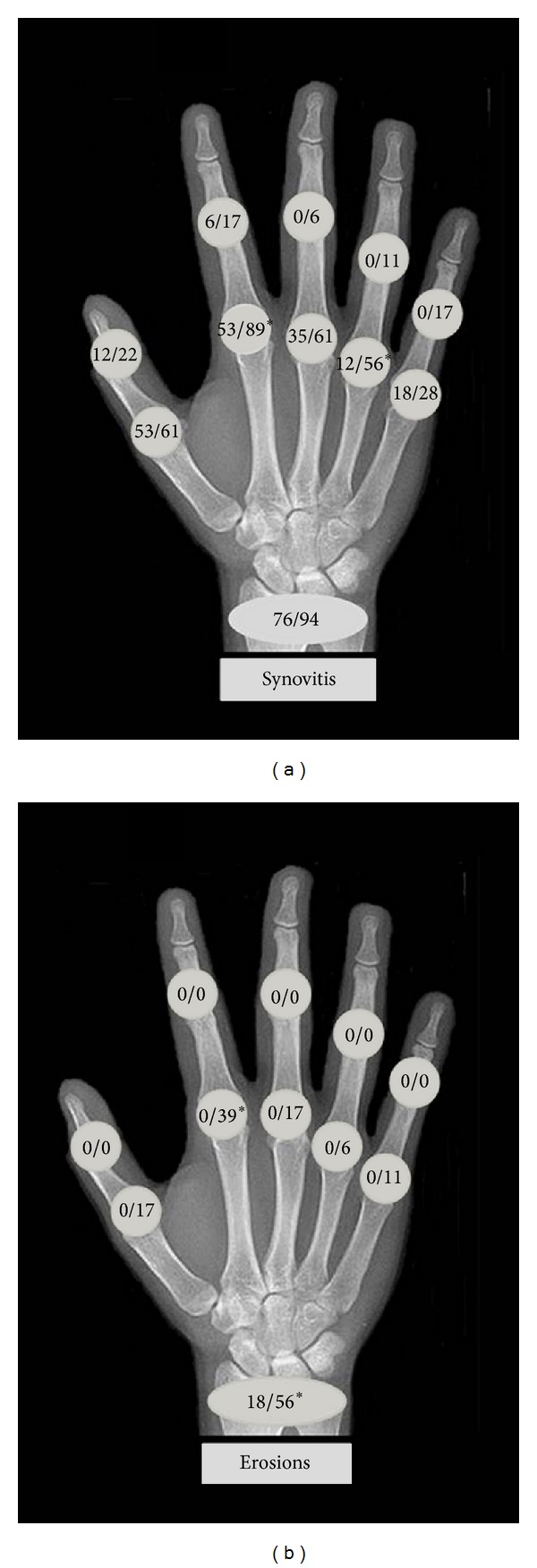
“Target area approach to arthritis” in the hands of patients with primary and secondary Sjögren's syndromes. The figure shows the frequency (percentage) of synovitis (a) and erosions (b). The upper digit corresponds to patients with primary Sjögren's syndrome, whereas the lower digit corresponds to patients with RA-associated Sjögren's syndrome. *P < 0.05.

Twenty-eight joints were evaluated in each patient, for a total of 476 scanned joints both in patients with pSS and no-Sjögren individuals and of 504 joints in those with sSS. Interobserver agreement was *κ* = 0.78 for synovitis and *κ* = 0.85 for erosions. The “target area approach to arthritis” in hands is shown in the Figure 1.

**Figure 2:**
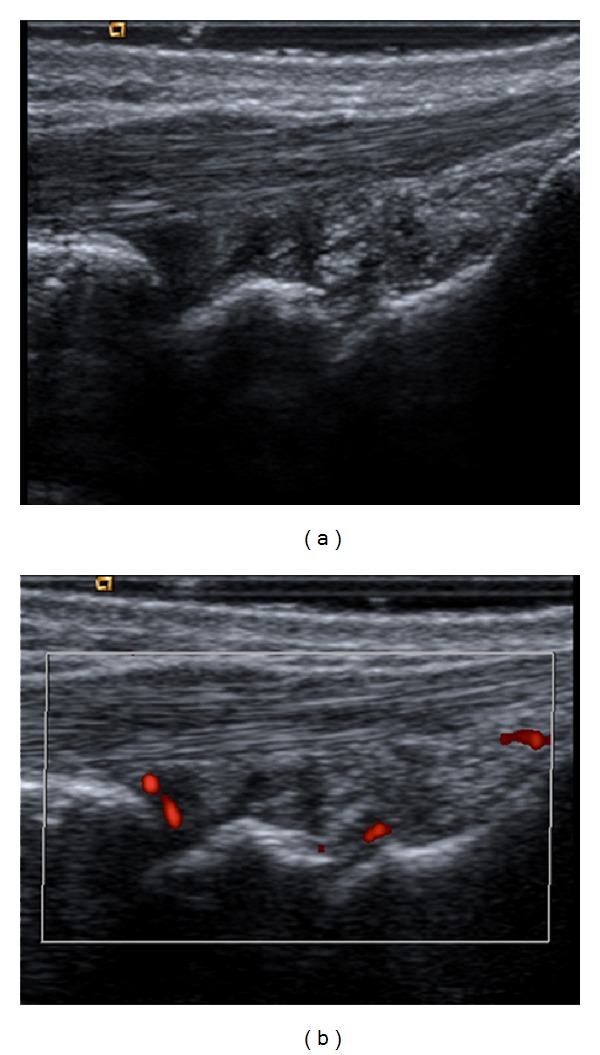
Synovitis. Radiocarpal and midcarpal joint recesses in grayscale (a) and power Doppler (b) longitudinal scans. Images show joint cavity widening, synovial proliferation and joint effusion (mixed pattern of synovitis), and intra-articular power Doppler signals in a patient with primary Sjögren's syndrome.

Synovitis (Figure 2) was found in 59 of 476 (12%) joints in pSS, whereas it was found in 110 (22%) of 504 joints in patients of sSS (Table 2). Furthermore, synovitis was the most common US finding in the small joints of the hands (MCP and PIP joints) in both groups, although it was more frequent in sSS (89 versus 53% at 2nd MCP joints; *P* = 0.02, and 56 versus 12% at 4th MCP joints; *P* = 0.01). In addition, synovitis was more frequent in the elbows of patients with sSS with respect to those with pSS (39 versus 6%; *P* = 0.04). No differences were observed regarding the presence of synovitis at knee, wrist, and ankle joints between groups. Synovitis was more commonly found in the 1st MCP joints of patients with pSS than in controls (53 versus 6%; *P* = 0.006), although this was similar in all the other articular areas scanned. Intra-articular PD signals (Table 3) were found in 23 joints in patients with sSS, while these were present in only four joints of patients with pSS (5 versus 0.8%; *P* = 0.0004) and in six joints of no-Sjögren individuals (1.2%; *P* = ns versus pSS patients).

**Figure 3:**
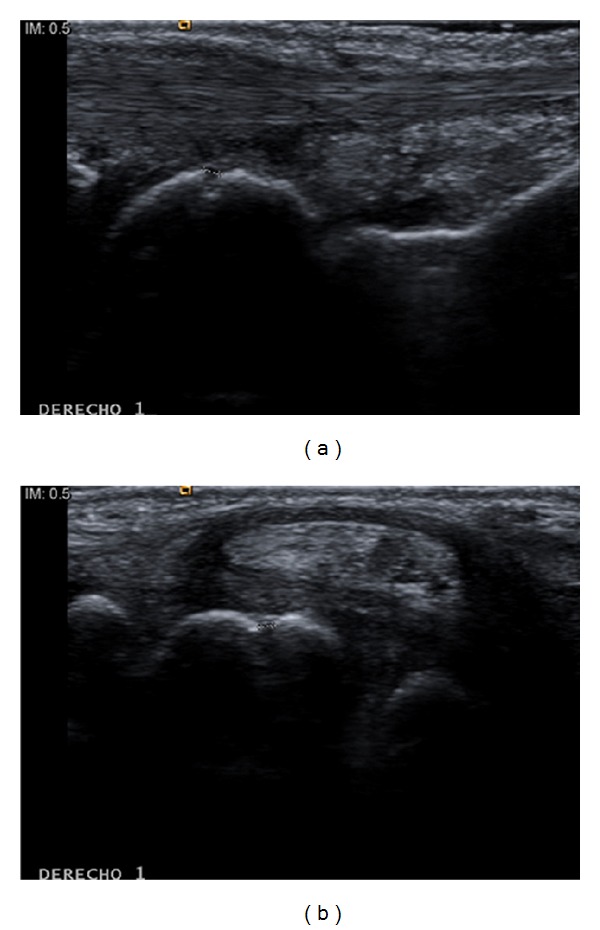
Bone erosion. Cortical defect (0.9 mm) on the semilunar bone (*os lunatum*) in longitudinal (a) and transverse (b) scans in a patient with primary Sjögren's syndrome.

A total of 28 erosions (Figure 3) were found in patients with sSS (Table 4), whereas only three were found in those with pSS (6 versus 0.6%; *P* = 0.0002). In the small finger joints, at least one erosion was found in 15 patients with sSS, while this was not observed in patients with pSS (83 versus 0%; *P* < 0.001). The 2nd MCP joint (39%) was the most common site for erosions in sSS, followed by the 1st and 3rd MCP joints (17% for each). At the wrist level, US showed erosions in 10 patients with sSS and in three patients with pSS (56 versus 18%; *P* = 0.03). It is noteworthy that erosions were not found in the X-rays of patients with pSS. Interestingly, one patient with pSS and wrist erosions was positive for anti-CCP antibodies and RF. No erosions were observed in individuals from the control group. 

The distribution pattern of synovitis, PD signals, and bone erosions was found to be symmetrical between each side of the body in patients with pSS and in those with sSS (data not shown).

Of note, no associations between the presence of each US sign and different laboratory data including antibodies, RF, and C-reactive protein concentrations were found in the group of patients with pSS. 

Finally, US was found to be useful to discriminate between patients with sSS and with pSS. Indeed, detecting US erosions in the 2nd MCP joint showed 28.8% sensitivity and 100% specificity for distinguishing the patients with RA-associated Sjögren's syndrome from those with the primary form of the syndrome, while these figures were 83.3 and 82.3%, respectively, for the presence of erosions in at least one (any) of the anatomical areas scanned in this study. In comparison, the presence of circulating anti-CCP antibodies showed 72.2% sensitivity and 94.1% specificity.

## 4. Discussion

Recent advances have placed US as a sensitive and reliable imaging method to assess morphostructural changes of joints, even in subclinical phases of diverse inflammatory and autoimmune diseases [9, 24, 25]. To the best of our knowledge, this is the first comprehensive study aimed at demonstrating the US pattern of joint involvement in pSS by means of a “target area approach to arthritis” [26].

The first US study aimed at characterizing joint involvement in pSS was performed in the knees of 60 patients by Iagnocco and colleagues [18]. This seminal report demonstrates the common occurrence of slight synovial thickness and joint effusion in the knees of patients with pSS while denying the existence of bone erosions. Our results are in line because we found a 76% frequency of either synovial thickening or joint effusion in the knees of patients with pSS, in the absence of PD signals or erosions. Additionally, we have included a wide range of small and large joints, which allows us to confirm the presence of polyarticular involvement in pSS.

In 2009, Riente and colleagues conducted a sonographic evaluation of the hand joints in 48 patients with pSS [17]. Only patients negative to anti-CCP antibodies were included. These authors found evidence of inflammatory arthritis in nine patients and bone erosions in MCP and/or PIP joints in six of these (12% prevalence). In the present study, erosions were detected in the radiocarpal and midcarpal joints of three patients with pSS (18% prevalence). In analogy with our results, Iagnocco and colleagues recently analyzed US changes in the hands and wrists of 32 patients with pSS [16]. Of a total of 960 joints evaluated, synovial proliferation was found in 5.1%, joint effusion in 3.4%, PD signals in 2%, and bone erosions in 0.2% (erosions were present in the wrists of a single patient with pSS.) The occurrence of bone erosions at the wrist level, but not in other anatomic areas, has been previously described in a study based on magnetic resonance imaging [27]. These results suggest that the distribution of hands joint involvement in pSS is similar to that found in patients with RA, namely, common involvement of MCP, PIP, and wrist joints, while distal IP joints are in their majority spared.

Synovium is the target of several inflammatory conditions such as RA, SLE, or pSS, although erosive structural damage is rare in arthritides other than RA. This concept has been challenged in the last decade by the advent of advanced imaging techniques to the field of rheumatology. For instance, Wright and colleagues detected bone erosions, by US, in 8 of 47 SLE patients at 2nd and 3rd MCP joints; of note, hand X-rays showed no erosions in three of them [14]. Erosions at the wrists also are demonstrated by US in some patients with SLE, often in association with joint synovitis and tenosynovitis [28]. Whether erosions found in patients with pSS result from osteoclast activation with active bone resorption (as seen in RA) or these result from tendon traction on demineralized bone with a subsequent ischemic bone resorption (as observed in systemic sclerosis) remains to be elucidated [29]. When occur, erosions appear to be related with a particular immune and pathogenetic background [30]. Indeed, some patients with SLE may develop erosive arthritis (a rare syndrome often called “rhupus”) in the context of circulating anti-CCP antibodies and alleles of MHC class II molecules including HLA-DRB1 and -DQB1 [31, 32]. Similar results have been described for psoriatic arthritis [33]. In terms of pSS, the prevalence of anti-CCP antibodies ranges from 3–10%, but their clinical significance remains uncertain [34–37]. Several cohort studies are in agreement with the fact that patients with pSS who are anti-CCP positive did not differ from those who are anti-CCP negative in terms of demographics, extraglandular involvement, synovitis, or other immunologic hallmarks [34, 35]. Contrariwise, other studies have found an association between anti-CCP antibodies and nonerosive synovitis [36, 37]. In the present study, the presence was noteworthy of erosive arthritis in three patients with pSS, one of these also having anti-CCP antibodies. Demonstration of bone erosions by advanced imaging methods in patients with pSS and circulating anti-CCP antibodies may lead to an oversimplification in the form of classifying these patients as having RA and sSS. We, however, believe that this assumption could be a *cum hoc ergo propter hoc* fallacy. Consequently, accurate characterization on the occurrence of bone erosions in pSS and their relationship with anti-CCP antibodies needs to be conducted.

According to the so-called “target area approach to arthritis” [26], analysis of the morphology of abnormalities and distribution of changes by US show that pSS often leads to polyarticular, bilateral, symmetrical, and predominantly nonerosive arthritis. In addition, this typically affects both small and medium-to-large joints, as noted in Tables 2, 3, and 4 as well as in the Figure 1.

We are aware that our study has limitations, including the lack of validation for morphostructural changes by advanced imaging techniques, the absence of longitudinal followup to determine whether patients with pSS and erosions could have later developed unequivocal RA, the small number of patients included, and the fact that a dichotomous (presence/absence) descriptive report of US findings was used instead of a true morphometric assessment. Further studies in larger cohorts are necessary to confirm our data, although these are provocative in nature.

## 5. Conclusions

The present US study provides useful information on the pattern and distribution of joint changes in pSS, which is predominantly polyarticular, bilateral, and symmetrical in distribution. Synovitis is the most frequently observed sign, especially in the small finger hand joints, the wrists, and the knees; however, bone erosions also may occur.

The presence of synovitis by US is not an accurate element for discriminating between RA-associated sSS and the primary form of the syndrome, although demonstration of bone erosions in the MCP joints seems to be useful.

## Figures and Tables

**Table 1:** Characteristics of patients with primary and secondary Sjögren's syndromes as well as no-Sjögren controls. Data are presented in median (range) unless otherwise specified.

	Primary Sjögren's syndrome (*n* = 17)	Secondary Sjögren's syndrome (*n* = 18)	*P* value**	No-Sjögren controls (*n* = 17)	*P* value***
Female, *n* (%)	17 (100)	17 (94)	ns	17 (100)	ns
Age, years	58 (32–83)	61 (40–77)	ns	58 (32–83)	ns
SS duration, years	3 (1–18)	9 (1–30)	0.006	—	—
Xerostomia, *n* (%)	17 (100)	18 (100)	ns	—	—
Keratoconjunctivitis, *n* (%)	17 (100)	18 (100)	ns	—	—
Salivary gland biopsy*, + (%)	6/8 (75)	4/6 (67)	ns	—	—
CRP, mg/L	1.9 (0.3–15.1)	7.7 (1.1–44.3)	ns	1.1 (0.1–8.2)	ns
RF, + (%)	14 (82)	18 (100)	ns	1 (6%)	<0.0001
Anti-CCP, + (%)	1 (6)	13 (72)	<0.0001	0	ns
ANA, + (%)	16 (94)	18 (100)	ns	0	<0.0001
Anti-Ro/SSA, + (%)	10 (59)	2 (11)	0.004	0	0.0003
Anti-La/SSB, + (%)	6 (35)	0	0.007	0	0.01

*More than one focus of lymphocytic infiltration in minor salivary tissue.

**Differences between patients with primary and secondary Sjögren's syndromes.

***Differences between patients with primary Sjögren's syndrome and no-Sjögren controls.

Abbreviations: SS: Sjögren's syndrome; CRP: C-reactive protein; RF: rheumatoid factor; anti-CCP: antibodies against cyclic citrullinated peptides; ANA: antinuclear antibodies.

**Table 2:** Presence of synovitis in the study groups.

Joint	Primary Sjögren's syndrome (*n* = 17)	Secondary Sjögren's syndrome (*n* = 18)	*P* value*	No-Sjögren controls (*n* = 17)	*P* value**
1st MCP	9	11	ns	1	0.006
2nd MCP	9	16	0.02	4	ns
3rd MCP	6	11	ns	4	ns
4th MCP	2	10	0.01	4	ns
5th MCP	3	5	ns	4	ns
1st IP	2	4	ns	3	ns
2nd PIP	1	3	ns	0	ns
3rd PIP	0	1	ns	1	ns
4th PIP	0	2	ns	3	ns
5th PIP	0	3	ns	0	ns
Wrist	13	17	ns	13	ns
Elbow	1	7	0.04	1	ns
Knee	13	17	ns	15	ns
Ankle	0	3	ns	3	ns

*Differences between patients with primary and secondary Sjögren's syndromes.

**Differences between patients with primary Sjögren's syndrome and no-Sjögren controls.

Abbreviations: MCP: metacarpophalangeal joints; IP: interphalangeal joint; PIP: proximal interphalangeal joints; PD: power Doppler signal.

**Table 3:** Presence of power Doppler signals in the study groups.

Joint	Primary Sjögren's syndrome (*n* = 17)	Secondary Sjögren's syndrome (*n* = 18)	*P* value*	No-Sjögren controls (*n* = 17)	*P* value**
1st MCP	0	1	ns	0	ns
2nd MCP	0	3	ns	0	ns
3rd MCP	0	4	ns	0	ns
4th MCP	0	2	ns	1	ns
5th MCP	0	1	ns	0	ns
1st IP	0	0	ns	0	ns
2nd PIP	0	0	ns	0	ns
3rd PIP	0	0	ns	0	ns
4th PIP	0	0	ns	0	ns
5th PIP	0	0	ns	0	ns
Wrist	2	8	ns	3	ns
Elbow	1	0	ns	0	ns
Knee	1	0	ns	2	ns
Ankle	0	0	ns	0	ns

*Differences between patients with primary and secondary Sjögren's syndromes.

**Differences between patients with primary Sjögren's syndrome and no-Sjögren controls.

Abbreviations: MCP: metacarpophalangeal joints; IP: interphalangeal joint; PIP: proximal interphalangeal joints; PD: power Doppler signal.

**Table 4:** Presence of bone erosions in the study groups.

Joint	Primary Sjögren's syndrome (*n* = 17)	Secondary Sjögren's syndrome (*n* = 18)	*P* value*	No-Sjögren controls (*n* = 17)	*P* value**
1st MCP	0	3	ns	0	ns
2nd MCP	0	7	0.007	0	ns
3rd MCP	0	3	ns	0	ns
4th MCP	0	1	ns	0	ns
5th MCP	0	2	ns	0	ns
1st IP	0	0	ns	0	ns
2nd PIP	0	0	ns	0	ns
3rd PIP	0	0	ns	0	ns
4th PIP	0	0	ns	0	ns
5th PIP	0	0	ns	0	ns
Wrist	3	10	0.03	0	ns
Elbow	0	1	ns	0	ns
Knee	0	1	ns	0	ns
Ankle	0	0	ns	0	ns

*Differences between patients with primary and secondary Sjögren's syndromes.

**Differences between patients with primary Sjögren's syndrome and no-Sjögren controls.

Abbreviations: MCP: metacarpophalangeal joints; IP: interphalangeal joint; PIP: proximal interphalangeal joints; PD: power Doppler signal.

## Abbreviations

pSS: Primary Sjögren's syndrome
RA: Rheumatoid arthritis
anti-CCP: Antibodies against cyclic citrullinated antibodies
MHC: Major histocompatibility complex
US: Ultrasound
SLE: Systemic lupus erythematosus
PD: Power Doppler
sSS: Secondary Sjögren's syndrome
RF: Rheumatoid factor
ANA: Antinuclear antibodies
MCP: Metacarpophalangeal
IP: Interphalangeal
PIP: Proximal interphalangeal
OMERACT: Outcome Measures in Rheumatology.
